# Efficacy of acupuncture for lumbar disc herniation: changes in paravertebral muscle and fat infiltration – a multicenter retrospective cohort study

**DOI:** 10.3389/fendo.2024.1467769

**Published:** 2024-11-06

**Authors:** Liang Yan, Jiliang Zhang, Xianliang Wang, Qinming Zhou, Jingdong Wen, Haihong Zhao, Kai Guo, Jianhua Zeng

**Affiliations:** ^1^ Department of Orthopaedic Surgery, The Third Hospital of Nanchang, Nanchang People's Hospital, Nanchang, Jiangxi, China; ^2^ Department of Rehabilitation Medicine, Xingguo Hospital Affiliated with Gannan Medical University, Ganzhou, Jiangxi, China; ^3^ Department of Acupuncture Rehabilitation, Ganzhou Hospital of Traditional Chinese Medicine, Ganzhou, Jiangxi, China; ^4^ Department of Orthopaedic Surgery, Ganxian District Hospital of Traditional Chinese Medicine, Ganzhou, Jiangxi, China; ^5^ Department of Traditional Chinese Medicine, Ganzhou Hospital of Guangdong Provincial People’s Hospital, Ganzhou City Hospital, Ganzhou, Jiangxi, China; ^6^ Department of Spine Surgery, Shanghai East Hospital, School of Medicine, Tongji University, Shanghai, China

**Keywords:** acupuncture, lumbar disc herniation, paraspinal muscles, fat infiltration, VAS Score

## Abstract

**Objective:**

This study seeks to elucidate the dynamic alterations in the multifidus, erector spinae, and psoas major muscles, along with their fatty infiltration, in patients diagnosed with lumbar disc herniation treated through acupuncture. Concurrently, the Visual Analogue Scale (VAS) and Japanese Orthopedic Association (JOA) scores are employed to evaluate modifications in lumbar and leg pain and the enhancement in lumbar functionality.

**Methods:**

A retrospective multi-center cohort study enrolled 332 adult LDH patients. Participants were divided into acupuncture and rehabilitation therapy groups. The acupuncture cohort received targeted treatments at specific acupuncture points, while the rehabilitation group received traditional rehabilitative therapy. Magnetic Resonance Imaging (MRI) gauged muscle cross-sectional areas (Sm, Se, Sp) and their ratios to vertebral area (Sm/Sv, Se/Sv, Sp/Sv), and fatty infiltration areas (Sfm, Sfe, Sfp) and their ratios (Sfm/Sv, Sfe/Sv, Sfp/Sv). Pain and function were assessed using Visual Analogue Scale (VAS) and Japanese Orthopedic Association (JOA) scores pre-treatment, 2-weeks, and 3-months post-intervention.

**Results:**

A total of 332 patients were enrolled for analysis. Post-treatment, the acupuncture group exhibited increased Sm, Se, Sp and their ratios and reduced fatty infiltration areas and their ratios (P<0.05) compared to rehabilitation. Both treatments decreased VAS scores and enhanced JOA scores at both intervals (P<0.05). Intriguingly, no significant disparities were observed between the acupuncture and rehabilitation groups concerning pain and JOA scores at the 2-week follow-up (p>0.05); however, 3 months post-treatment, the acupuncture group significantly outperformed the rehabilitation group in both pain and JOA scores (p<0.05).

**Conclusion:**

This study demonstrates that acupuncture treatment is significantly more effective than traditional rehabilitation therapy in improving paraspinal muscle function, reducing muscle fat infiltration, and alleviating lumbar and leg pain in patients with lumbar disc herniation (LDH). Specifically, acupuncture significantly increases the cross-sectional areas (Sm, Se, Sp) of the paraspinal muscles and reduces muscle fat infiltration, showing superior long-term results in pain relief and functional improvement. Future research should further explore the long-term effects of acupuncture on the function and structure of paraspinal muscles, assess its potential in preventing the recurrence of LDH, and delve deeper into how acupuncture affects paraspinal muscles at the molecular level, to better understand its therapeutic mechanisms and enhance its clinical application.

## Introduction

Lumbar disc herniation (LDH) is a prevalent degenerative disorder characterized by lower back pain, radiating pain in the lower extremities, and cauda equina syndrome. It stands as a common etiology for lower back discomfort, neurological dysfunction, and pain in the buttock/leg region (1), Consequently, LDH can significantly compromise the quality of life of affected individuals, posing a considerable economic strain on both families and the broader healthcare system (2). Epidemiological studies suggest that globally, around 80% of individuals will experience low back pain at least once in their lifetimes, with a significant portion attributed to disc herniation (3–5). Owing to shifts in lifestyle and work patterns, such as prolonged sitting and overweight issues, the prevalence of this ailment has been escalating among younger populations (6).

In clinical settings, it is often observed that radiological findings do not consistently align with the clinical presentation of patients. Notably, certain patients with MRI/CT scans indicating disc protrusion or extrusion may only exhibit mild symptoms, while others with minimal herniation present with pronounced manifestations (5). This suggests that symptoms stemming from LDH aren’t solely associated with spinal canal compression and inflammation. A comprehensive prospective study illuminated various determinants of early disc herniation resorption, identifying that nearly a quarter of LDH patients experienced early absorption (7). In recent years, the role of paraspinal muscles in maintaining spinal stability has garnered increasing attention. As one of the most vital muscles surrounding the spine, paraspinal muscles are essential for preserving spinal stability and dynamic function (8). The paraspinal group includes the multifidus and the erector spinae muscles, the latter of which is further divided into the spinalis, longissimus, and iliocostalis (9). The quantity and quality of these muscles directly impact lumbar health. The quantity of paraspinal muscles is typically quantified by measuring the cross-sectional area (CSA) of the multifidus, erector spinae, and iliocostalis via MRI, which helps assess the extent of muscle atrophy (10). The quality of paraspinal muscles involves evaluating muscle composition, particularly the proportion of fatty tissue within the muscles assessed through MRI imaging, reflecting the health and functional status of the muscles (11). Changes in the quality and quantity of paraspinal muscles have significant clinical implications for lumbar health (8, 12): Specifically, the atrophy of muscles, particularly the multifidus, is associated with spinal instability, potentially leading to or exacerbating lower back pain and spinal functional impairments (12). Moreover, the degeneration of paraspinal muscles, such as fat infiltration and muscle atrophy, is often linked to symptoms of lower back pain (11, 13). The ability to predict and treat these symptoms is crucial clinically, as strengthening paraspinal muscles can effectively alleviate pain and enhance functionality. Therefore, a deeper understanding of changes in paraspinal muscles can assist physicians in developing more effective rehabilitation plans, including targeted physical therapy and exercises to restore or improve muscle function. In young LDH patients with unilateral neurological symptoms, bilateral MF atrophy is more likely to induce lower back pain (14). When paraspinal muscles undergo atrophy, stiffness, or dysregulation, lumbar stability is compromised, leading to uneven stress distribution on the intervertebral discs and disruption of the intervertebral cushioning system, subsequently impacting the disc’s workload. A meta-analysis focusing on muscle fiber size, distribution, and overall muscle lateral differences highlighted that LDH patients had more pronounced fat infiltration and atrophy in the MF compared to a control group. These alterations could further influence post-treatment pain and recovery outcomes (15). Another prospective study using MRI assessed isokinetic back muscle strength and both the quantity and quality of paraspinal muscles. The findings indicated that degeneration of paraspinal muscles might expedite lumbar deterioration (16). Factors such as prolonged sitting, excessive loading, and poor posture contribute to lumbar muscle strain, leading to functional imbalances and a reduction in spinal stability. In summary, assessing the quality and quantity of paraspinal muscles is vital for diagnosing and treating muscle functional degradation in patients with lumbar disc herniation (15). Hence, the health status of lumbar paraspinal muscles, such as asymmetry in muscle area, atrophy, fat infiltration, and functional imbalances, could be pivotal factors influencing the symptoms of LDH.

While ultrasonography and CT scans have traditionally been employed to assess the morphology of paraspinal muscles (17–19), these modalities exhibit reduced capacity to differentiate muscle tissues and are prone to larger errors. In contrast, MRI emerges as a superior diagnostic tool due to its non-invasive nature, repeatability, and high-resolution imaging capabilities. MRI not only enables precise determination of vertebral segment levels and identification of intraspinal pathological conditions but also enables accurate measurement of the density and area of paraspinal muscles and the extent of intermuscular fat infiltration (20), Clinically, MRI is extensively utilized for the observation and diagnosis of neurological and musculoskeletal soft tissues.

While the majority of current treatments for LDH remain non-surgical, studies indicate that appropriate non-operative interventions can alleviate symptoms in 85%-90% of patients. These approaches primarily aim to enhance paraspinal muscle function and regulate spinal biomechanical balance. Prevalent clinical treatments encompass conservative therapies, minimally invasive interventions, and surgical procedures (21). Among these, conservative therapies like acupuncture, physical therapy, and exercise rehabilitation have been widely adopted in clinical practice. Notably, acupuncture has been demonstrated to effectively treat musculoskeletal soft tissue injuries (22–24), By stimulating structures such as myofascia, tendons, and muscles, it can enhance local micro-circulation and blood supply, and has been established as a safe procedure (25, 26). Compared to other conservative treatments, acupuncture has shown unique advantages in improving the quality of paravertebral muscles and alleviating pain (27). The choice of acupuncture as the treatment modality in our study is based on the traditional applications of Traditional Chinese Medicine (TCM) theory, as well as the effects demonstrated in modern research (28–30). Specific acupuncture points have been shown to directly improve lower back and leg pain by regulating the natural healing processes of lumbar disc herniation and pain perception (31). Furthermore, rehabilitation therapy, diverse in methods and easy to administer, has been widely utilized in the clinic, with satisfactory short-term results (32, 33), Thus, in this study, we chose rehabilitation therapy as the comparative treatment.

While acupuncture has shown significant efficacy in treating lumbar disc herniation, scant research has employed MRI to assess the specific impacts of acupuncture on paraspinal muscles and fat infiltration. In this vein, our study aims to delve into the effects of acupuncture on the structural changes of paraspinal muscles and fat infiltration in patients with LDH using MRI. Concurrently, we will evaluate the influence of acupuncture on patients’ VAS and JOA scores, comparing these outcomes to those undergoing rehabilitation therapy. In this study, we hypothesize that acupuncture treatment can significantly improve the cross-sectional area of paraspinal muscles (Sm, Se, Sp) and reduce muscle fat infiltration in patients with lumbar disc herniation (LDH). Through these improvements, acupuncture is expected to alleviate lumbar and leg pain, thereby enhancing overall quality of life. Compared to conventional rehabilitation therapy, we predict that acupuncture will show significant increases in the cross-sectional area of paraspinal muscles, significant reductions in muscle fat infiltration, and significant improvements in Visual Analogue Scale (VAS) and Japanese Orthopaedic Association (JOA) scores at 2 weeks and 3 months post-treatment. Specifically, acupuncture is expected to exhibit superior long-term effects in pain relief and functional improvement. Through this research, we aspire to gain a more comprehensive understanding of the underlying mechanisms of acupuncture in the context of LDH.

## Materials and methods

We conducted a multi-center retrospective cohort study across 6 hospitals in China, in strict adherence to the principles of the Declaration of Helsinki. The research protocol received thorough review and approved by the ethics committees of several respected institutions: Shanghai East Hospital, Ganzhou Hospital of Traditional Chinese Medicine in Jiangxi Province, Ganzhou Hospital of Guangdong Provincial People’s Hospital, Xingguo Hospital affiliated with Gannan Medical College, The Third Hospital of Nanchang, and Ganxian District Hospital of Traditional Chinese Medicine, Jiangxi Province. All participants, after receiving comprehensive education regarding the study, provided their informed consent in writing. They were also informed of their right to withdraw from the study at any time without repercussion. From June 2020 to June 2023, we gathered 332 patients diagnosed with Lumbar Disc Herniation (LDH) from acupuncture outpatient rehabilitation departments and rehabilitation medicine center wards. The cohort was divided into two groups: 166 patients received acupuncture treatment and 166 underwent rehabilitation therapy. The study collected data on age, gender, Body Mass Index (BMI), medical history, clinical diagnosis, affected areas, and side of pain. Additionally, Japanese Orthopaedic Association (JOA) scores, Visual Analogue Scale (VAS) scores for back pain and leg pain were recorded both before and after the treatment to assess the therapeutic effects. The diagnostic criteria for LDH are based on the expert consensus and related literature of the 2014 revised Guidelines of the North American Spine Association (8, 14, 15, 34, 35). Inclusion Criteria: 1.Patients diagnosed with Lumbar Disc Herniation (LDH) without indications for surgical intervention. 2. Duration of the condition not exceeding three months, with no recurrent symptoms. 3. Age between 18 to 70 years, irrespective of gender. 4. Radiological findings consistent with the symptoms and signs of the corresponding spinal segment. 5. Patients who refuse pharmacological treatment and opt for acupuncture or rehabilitation therapy. Exclusion Criteria: 1. Patients exhibiting progressive symptoms of neurological damage. 2. Patients diagnosed with cauda equina syndrome. 3. Presence of lumbar instability, lumbar injury, or spinal tumors. 4. Symptoms and signs involving more than two vertebral segments. 5. Patients with systemic infectious diseases (such as osteomyelitis, systemic lupus erythematosus, ankylosing spondylitis, rheumatoid arthritis), severe hematologic diseases, infectious diseases, skin lesions, allergic constitution, or mental disorders. 6. Patients with severe heart disease, severe diabetes, or Parkinson’s disease, or other serious chronic conditions. 7. Patients non-compliant with treatment or follow-up procedures. We established strict screening criteria for participants, explicitly excluding those with chronic conditions such as heart disease, severe diabetes, or Parkinson’s disease that might affect the study outcomes. Throughout the study, we regularly monitored the patients’ blood pressure, blood sugar, and other relevant health indicators to ensure they remained within acceptable limits. By ensuring comparability in baseline characteristics such as age, gender, Body Mass Index (BMI), and duration of illness, we enhanced the scientific rigor and fairness of the research (36, 37). The work used the CONSORT reporting guidelines (38).

### Treatment method

**Figure 1:**
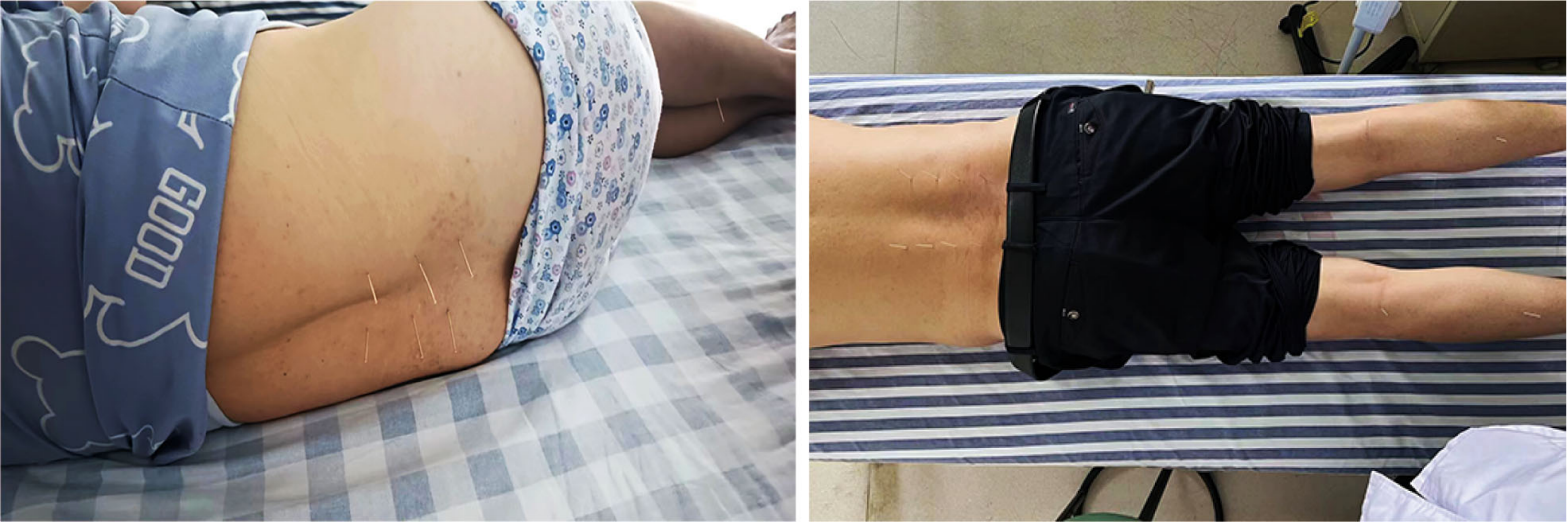
Acupuncture treatment diagram for patients with lumbar disc herniation.

**Figure 2:**
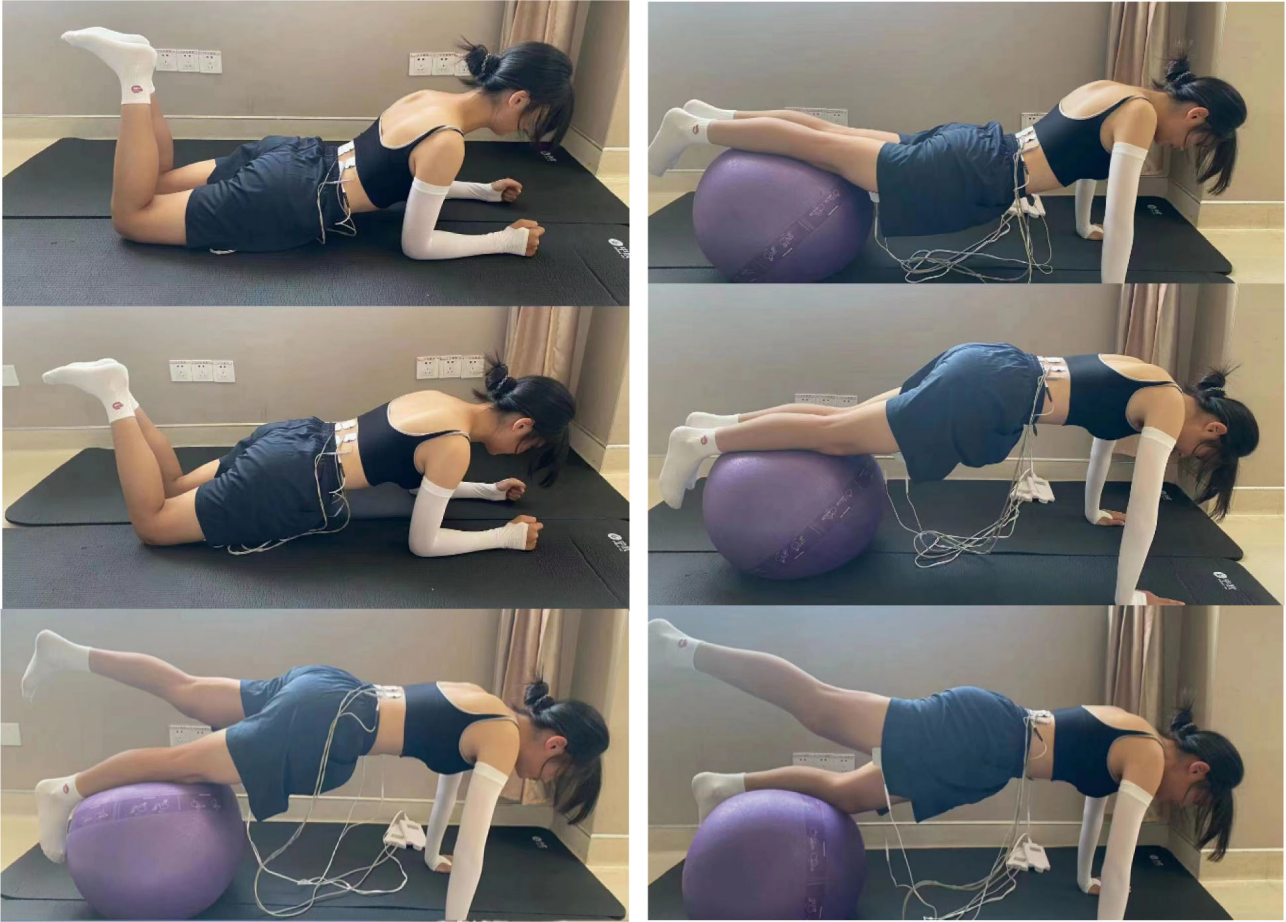
Rehabilitation Diagram: Plank, Bridge Exercise, and Iliopsoas Stretch.

In the acupuncture group, acupoints such as Shen Shu (BL 23), Yao Yang Guan (GV 3), Da Chang Shu (BL 25), Zhi Bian (BL 54), Wei Zhong (BL 40), Cheng Shan (BL 57), Yang Ling Quan (GB 34), and Kun Lun (BL 60) were selected for treatment. Prior to each needle insertion, the skin was thoroughly disinfected with alcohol. We employed fine needles of dimensions0.3 x 40mm(Ring handle needle 0197, Suzhou Hualun Medical Supplies Co., LTD, China), inserting them vertically into the skin until a sensation of ‘De Qi’ or the arrival of qi was achieved. Once the needles were accurately positioned into the designated acupoints, they were retained for 20 minutes (**Figure 1**). Treatments were administered daily for a continuous period of 20 days. Conversely, Patients in the rehabilitation treatment group underwent lumbar rehabilitation therapy (**Figure 2**), which included plank exercises, low frequency electron pulse therapy(G6805-2B,Shanghai Huayi Medical Instrument Co., LTD,China), and iliolumbar muscle stretching exercises. The intensity of the rehabilitation regimen was adjusted based on patient tolerance. Each session lasted 20 minutes, conducted once daily for a duration of 20 days. All lumbar MRI evaluations were performed using a 3.0T magnetic resonance imaging system(Siemens, Germany). The study involved T1-weighted and T2-weighted scans, with each type of scan calibrated for specific diagnostic requirements(PACS System, Fujifilm (China) Investment Co., LTD). Lumbar MRI examinations were conducted prior to treatment to establish baseline data, and then repeated three months after treatment to assess therapeutic outcomes.

### Outcome measurements and data collection

**Figure 3:**
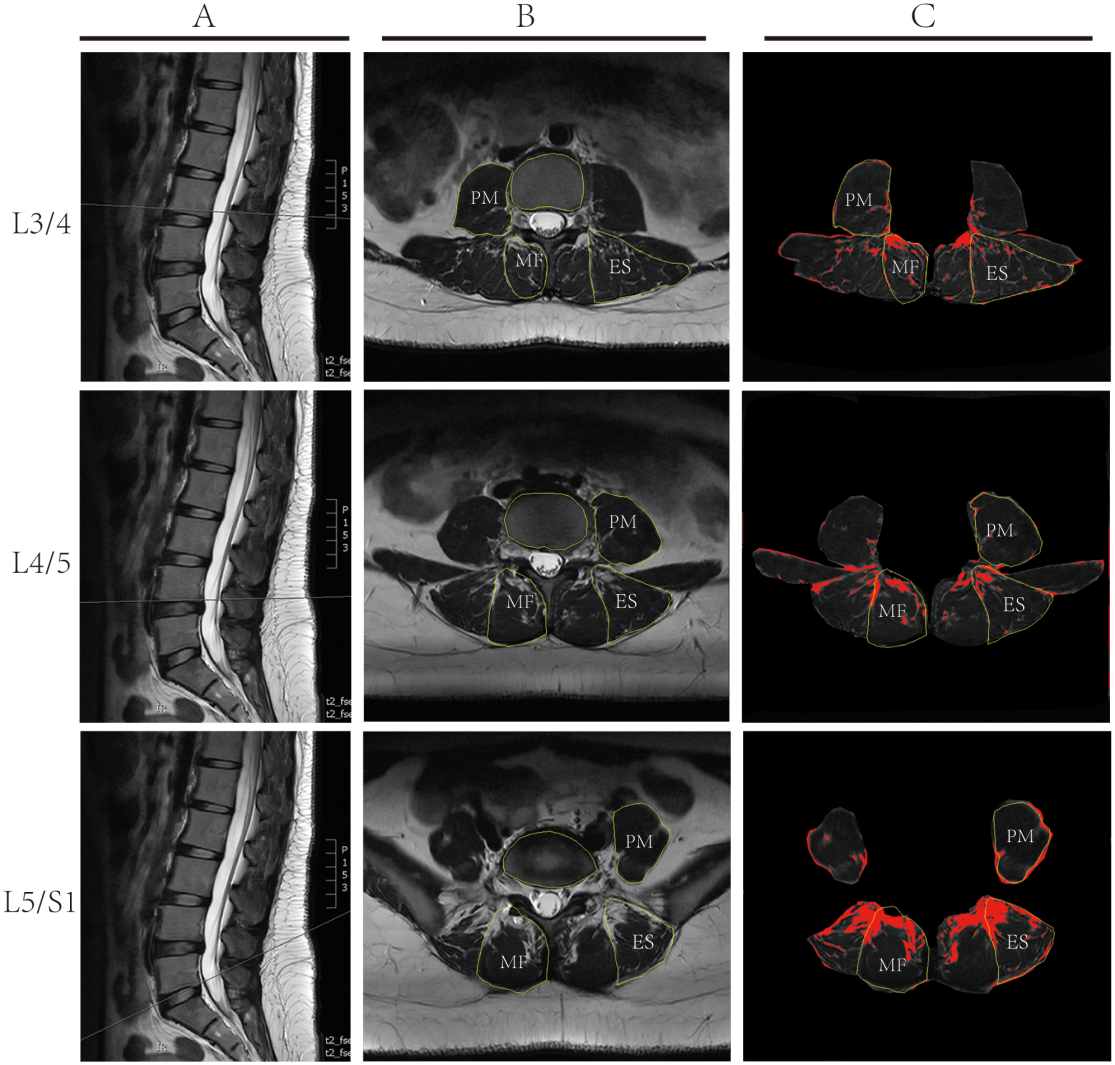
Illustrative Depiction of Lumbar MRI Measurement Techniques: **(A)** Sagittal T2 weighted MRI Imaging of the lumbar spine, depicting measurement planes for L3/4, L4/5, and L5/S1; **(B)** Cross-sectional Analysis using ImageJ software to delineate the multifidus muscle (MF), erector spinae (ES), psoas major muscle (PM), and vertebral contours. **(C)** Using ImageJ software and based on set thresholds, the fatty tissues within the muscles are marked in red, and the area of the fat is calculated.

In our study, we employed a comprehensive approach to meticulously evaluate the therapeutic outcomes of lumbar spine disorders. Our holistic assessment encompassed the cross-sectional areas (CSA) of MF, ES, and PM(*Sm, Se, and Sp* respectively), and their relative proportions to the vertebral area (*Sv*), given by ratios *Sm/Sv, Se/Sv*, and *Sp/Sv*. Additionally, we evaluated the fat infiltration areas (*Sfm, Sfe, Sfp*) and their respective ratios to the vertebral CSA (*Sfm/Sv, Sfe/Sv, Sfp/Sv*). Specifically, we chose T2-weighted images (T2WI) of the L3/4, L4/5, and L5/S1 vertebrae (**Figure 3A**) and utilized ImageJ software to measure the CSA of the aforementioned muscles and the vertebral body (**Figure 3B**). By configuring an appropriate threshold within the software, fatty tissue within the muscles was delineated in red (**Figure 3C**), facilitating the computation of its area. The area of each muscle and its fatty infiltration was calculated based on the average values obtained from both the left and right sides. Subsequently, we discerned the ratios of these muscle areas and fatty infiltration areas to the vertebral CSA, shedding light on the degree of paraspinal muscle atrophy and the extent of fatty infiltration. A smaller muscle ratio indicated pronounced muscle atrophy, whereas a higher fatty ratio denoted a substantial fatty infiltration within the muscles. Additionally, the study employed the Visual Analog Scale (VAS) and Japanese Orthopaedic Association (JOA) scores to assess pain and functional outcomes pre-treatment, at two weeks post-treatment, and after three months. The VAS scoring system was employed to gauge the effectiveness of the treatment, with a score of 0 representing an absence of pain and a score of 10 signifying the most intense pain. To provide a comprehensive overview of the patients’ quality of life, we also utilized the JOA scoring questionnaire. This tool examines the effects on various aspects such as pain intensity, daily care activities, weight lifting, ambulation, postural transitions between sitting and standing, and sleep patterns. A heightened JOA score denotes a more severe medical condition and evident functional limitations.

### Statistical analysis

Data analysis was performed using SPSS 20.0 statistical software. Quantitative data were expressed as mean ± standard deviation (X ± SD). For data conforming to normal distribution and homogeneity of variance, paired sample t-tests were used for within-group comparisons, and independent sample t-tests for between-group comparisons. For data with unequal variances, t’ tests were utilized. Count data were represented as counts and percentages (%), analyzed using the χ2 test. In chi-square tests, if the total sample size was n≥40 but the expected frequency in any cell 1≤T<5, a continuity correction chi-square test was applied; if T<1 or total sample size was n<40, Fisher’s exact test was used. All statistical tests were two-tailed, with a significance level set at P<0.05.

## Results

### Baseline data characteristics

In this study, we included a total of 332 patients with single-segment LDH. **Table 1** offers a comprehensive overview of the baseline characteristics of the patients. Participants were divided into the acupuncture group (n=166) and the rehabilitation treatment group (n=166). Out of the entire cohort, there were 160 males and 172 females. The average age for patients in the acupuncture group was 46.67 ± 15.29 years, while it was 45.61 ± 16.14 years for the rehabilitation group. The acupuncture group had a mean BMI of 28.84 ± 2.47kg/m^2^, while the rehabilitation group presented with a mean BMI of 23.41 ± 2.62kg/m^2^. The duration of the disease for the acupuncture and rehabilitation groups was 4.85 ± 2.51 months and 4.49 ± 2.16 months, respectively. Concerning the lumbar disc herniation segments, the acupuncture group comprised 20, 86, and 60 patients with L3/4, L4/5, and L5/S1 LDH, respectively. In contrast, the rehabilitation group had 27, 89, and 50 patients with L3/4, L4/5, and L5/S1 LDH, respectively. The distribution of left and right-sided pain in the acupuncture group was 84 and 82 cases, respectively, while in the rehabilitation group, it was 77 and 89 cases, respectively. Pre-treatment assessment scores for the acupuncture group were: lumbar VAS pain score of 4.18 ± 0.90, leg VAS pain score of 4.16 ± 0.98, and a JOA score of 19.01 ± 1.54. For the rehabilitation group, the scores were: lumbar VAS pain score of 4.10 ± 0.94, leg VAS pain score of 4.17 ± 1.05, and a JOA score of 18.86 ± 1.61. The two patient groups showed no statistically significant differences in terms of age, gender, Body Mass Index (BMI), affected segments, side of pain, pre-treatment JOA scores, and pre-treatment VAS scores for back and leg pain (all P values >0.05). This ensures the comparability of baseline data between the two groups prior to treatment.

**Table 1:** Baseline characteristics of lumbar disc herniation patients.

Parameter	Acupuncture Group	Rehabilitation Therapy Group	Statistical value	*p*-value
Age (years)	46.67 ± 15.29	45.61 ± 16.14	0.618	0.537
Gender (M/F)	86/80	74/92	1.737	0.187
BMI (kg/m^2^)	23.52 ± 2.27	23.41 ± 2.64	0.417	0.677
Duration (months)	4.85 ± 2.51	4.49 ± 2.16	1.408	0.160
Side (Left/Right)	84/82	77/89	0.591	0.442
Level (L3/4, L4/5, L5/S1)	20/86/60	27/89/50	2.003	0.367
JOA Score	19.01 ± 1.54	18.86 ± 1.61	0.871	0.385
Lumbar Pain VAS Score	4.18 ± 0.90	4.10 ± 0.94	0.836	0.404
Leg Pain VAS Score	4.16 ± 0.98	4.17 ± 1.05	0.108	0.914

M/F, Male/Female; VAS, Visual Analog Scale; JOA, Japanese Orthopedic Association.

### Analysis of paraspinal muscle CSA ratios before and after treatment

In this study, Multifidus (MF), Erector Spinae (ES), and Psoas Major (PM) were selected as research subjects, with vertebral body cross-sectional area (CSA) as a reference. T2-weighted imaging (T2WI) muscle CSA measurements at the lower edges of the L3, L4, and L5 vertebrae were conducted pre- and post-treatment, as shown in **Table 2**. The ratios of MF CSA to vertebral CSA *(Sm/Sv)* were calculated. Pre-treatment ratios at L3/4, L4/5, L5/S1 for acupuncture and rehabilitation groups were (0.74 ± 0.22, 0.71 ± 0.28, p=0.279), (0.82 ± 0.17, 0.80 ± 0.18, p=0.499), and (0.87 ± 0.17, 0.85 ± 0.18, p=0.551) respectively, showing no significant differences (P>0.05), ensuring comparability of baseline data. Post-treatment, the Sm/Sv ratios showed significant improvements in both groups: (0.80 ± 0.23, 0.73 ± 0.29, p=0.028), (0.87 ± 0.17, 0.83 ± 0.19, p=0.041), and (0.91 ± 0.16, 0.87 ± 0.19, p=0.041), with the acupuncture group exhibiting notably greater enhancements(P<0.05).Within-group analyses further revealed significant improvements post-treatment in both groups (P<0.001).For the ES CSA to vertebral CSA ratio *(Se/Sv)*, pre-treatment ratios at L3/4, L4/5, L5/S1 for acupuncture and rehabilitation groups were (1.09 ± 0.16, 1.06 ± 0.15, p=0.132), (1.10 ± 0.19, 1.11 ± 0.20, p=0.907), and (0.82 ± 0.22, 0.82 ± 0.23, p=0.947) respectively, indicating no significant differences (P>0.05) and similar baselines. Post-treatment ratios were (1.14 ± 0.15, 1.08 ± 0.15, p=0.002), (1.18 ± 0.20, 1.14 ± 0.21, p=0.043), and (0.89 ± 0.21, 0.84 ± 0.24, p=0.034), showing significant increases in both groups (P<0.05), with more pronounced elevation in the acupuncture group (P<0.05). Furthermore, within-group comparisons revealed that both the acupuncture and rehabilitation groups demonstrated significant improvements compared to their pre-treatment statuses (P<0.001).

**Table 2:** Primary outcomes for the acupuncture group and the rehabilitation therapy group *(SpA/Sv)*.

Variables	L3			L4			L5		
	Pre-treatment	Post-treatment	t	Pre-treatment	Post-treatment	t	Pre-treatment	Post-treatment	t
*S*_m_/*S*_v_									
Acupuncture	0.74 ± 0.22	0.80 ± 0.23	46.76^***^	0.82 ± 0.17	0.87 ± 0.17	141.50^***^	0.87 ± 0.17	0.91 ± 0.16	60.26^***^
Rehabilitation Therapy	0.71 ± 0.28	0.73 ± 0.29	31.23^***^	0.80 ± 0.18	0.83 ± 0.19	96.23^***^	0.85 ± 0.18	0.87 ± 0.19	36.45^***^
T	1.085	2.213^*^		0.677	2.051^*^		0.597	2.055^*^	
*P*	0.279	0.028		0.499	0.041		0.551	0.041	、
*Se/Sv*									
Acupuncture	1.09 ± 0.16	1.14 ± 0.15	118.20^***^	1.10 ± 0.19	1.18 ± 0.20	331.00^***^	0.82 ± 0.22	0.89 ± 0.21	110.63^***^
Rehabilitation Therapy	1.06 ± 0.15	1.08 ± 0.15	43.42^***^	1.11 ± 020	1.14 ± 0.21	43.22^***^	0.82 ± 0.23	0.84 ± 0.24	73.05^***^
T	1.511	3.149^**^		0.117	2.028^*^		0.067	2.218^*^	
*P*	0.132	0.002		0.907	0.043		0.947	0.034	
*Sp/Sv*									
Acupuncture	0.78 ± 0.18	0.82 ± 0.19	63.11^***^	1.13 ± 0.18	1.14 ± 0.19	117.84^***^	0.88 ± 0.18	0.90 ± 0.20	3.74^*^
Rehabilitation Therapy	0.77 ± 0.19	0.79 ± 0.18	72.7^***^	1.14 ± 0.22	1.15 ± 0.21	143.01^***^	0.85 ± 0.23	0.88 ± 0.24	4.08^*^
t	0.584	1.413		0.396	0.471		1.412	0.475	
*p*	0.56	0.159		0.693	0.638		0.159	0.635	

*Sv*, Vertebral Area; *SpA*, Paraspinal Muscle Cross-Sectional Area; *Sm*, Multifidus Cross-Sectional Area; *Se*, Erector Spinae Cross-Sectional Area; *Sp*, Psoas Major Cross-Sectional Area; *SpA/Sv*, Paraspinal Muscle Cross-Sectional Area to vertebral area ratio; *Sm/Sv*, Multifidus muscle cross-sectional Area to vertebral area ratio; *Se/Sv*, Erector spinae muscle cross-sectional Area to vertebral area ratio; *Sp/Sv*, Psoas major muscle cross-sectional Area to vertebral area ratio. ***p<0.001, **p<0.01, *p<0.05.

For the PM CSA to vertebral CSA ratio *(Sp/Sv)*, pre-treatment ratios at L3/4, L4/5, L5/S1 in the acupuncture and rehabilitation groups were (0.78 ± 0.18, 0.77 ± 0.19, p=0.56), (1.13 ± 0.18, 1.14 ± 0.22, p=0.693), and (0.88 ± 0.18, 0.85 ± 0.23, p=0.159) respectively, showing no significant differences between groups (P>0.05) and indicating similar baselines. Post-treatment ratios at L3/4, L4/5, L5/S1 were (0.82 ± 0.19, 0.79 ± 0.18, p=0.159), (1.14 ± 0.19, 1.15 ± 0.21, p=0.638), and (0.90 ± 0.20, 0.88 ± 0.24, p=0.635) respectively; there were no significant differences between groups post-treatment (P>0.05). However, within-group comparisons showed significant improvement in both groups compared to pre-treatment (P<0.05). In summary, acupuncture treatment showed superior results in improving the ratios of these three muscle CSA to the vertebral CSA compared to rehabilitation treatment. While both treatments notably ameliorated muscle atrophy in LDH patients, acupuncture therapy proved more effective in augmenting paravertebral muscle CSA, subsequently enhancing patients’ symptoms and quality of life.

### Analysis of fat infiltration in paraspinal muscles before and after treatment

In this study, we measured the fat tissue area and vertebral CSA of the ES, MF, and PM on the lumbar magnetic resonance T2WI images at the lower edges of the L3, L4, and L5 vertebrae. The ratios of each muscle’s fatty tissue area to vertebral CSA (Sfm/Sv, Sfe/Sv, Sfp/Sv) were computed. Pre-treatment, the acupuncture and rehabilitation groups at L3/4, L4/5, L5/S1 showed the following ratios for *Sfm/Sv*: (0.057 ± 0.021, 0.056 ± 0.018, p=0.739), (0.071 ± 0.026, 0.066 ± 0.023, p=0.07), (0.119 ± 0.032, 0.112 ± 0.033, p=0.058), for *Sfe/Sv*: (0.075 ± 0.020, 0.076 ± 0.019, p=0.765), (0.049 ± 0.025, 0.047 ± 0.018, p=0.506), (0.122 ± 0.031, 0.120 ± 0.026, p=0.725), and for *Sfp/Sv*: (0.029 ± 0.008, 0.030 ± 0.005, p=0.614), (0.026 ± 0.005, 0.025 ± 0.004, p=0.114), (0.029 ± 0.010, 0.030 ± 0.007, p=0.400), respectively, all with no statistical significance (P>0.05) (**Table 3**), ensuring baseline comparability. Post-treatment, the acupuncture and rehabilitation groups at L3/4, L4/5, L5/S1 showed the following ratios for *Sfm/Sv*: (0.047 ± 0.021, 0.053 ± 0.017, p=0.012), (0.049 ± 0.027, 0.055 ± 0.022, p=0.044), (0.089 ± 0.033, 0.104 ± 0.032, p<0.001); for *Sfe/Sv*: (0.040 ± 0.022, 0.063 ± 0.018, p<0.001), (0.035 ± 0.024, 0.041 ± 0.019, p=0.017), (0.095 ± 0.032, 0.102 ± 0.025, p=0.033); and for *Sfp/Sv*: (0.024 ± 0.008, 0.026 ± 0.004, p=0.016), (0.021 ± 0.005, 0.022 ± 0.004, p=0.013), (0.025 ± 0.010, 0.027 ± 0.006, p=0.011). The *Sfm/Sv*, *Sfe/Sv*, *and Sfp/Sv* for MF, ES, and PM at L3/4, L4/5, and L5/S1 decreased significantly compared to pre-treatment (P<0.05). This indicates that acupuncture treatment demonstrates evident efficacy in reducing muscular fat infiltration. In contrast, the ratio changes in the rehabilitation group were not as pronounced as those in the acupuncture group, indicating that the therapeutic effects of rehabilitation were not as significant as those of acupuncture (P<0.05).Within-group comparisons showed significant improvements in both groups compared to pre-treatment (P<0.001).

**Table 3:** Primary outcomes for the acupuncture group and the rehabilitation therapy group (*Sf/Sv*).

Variables	L3			L4			L5		
	Pre-treatment	Post-treatment	t	Pre-treatment	Post-treatment	t	Pre-treatment	Post-treatment	T
*Sfm/S*_v_									
Acupuncture	0.057 ± 0.021	0.047 ± 0.021	102.13^***^	0.071 ± 0.026	0.049 ± 0.027	58.804^***^	0.119 ± 0.032	0.089 ± 0.033	249.86^***^
Rehabilitation Therapy	0.056 ± 0.018	0.053 ± 0.017	38.13^***^	0.066 ± 0.023	0.055 ± 0.022	150.07^***^	0.112 ± 0.033	0.104 ± 0.032	50.49^***^
t	0.333	2.534^*^		1.820	2.026^*^		1.904	3.955^***^	
*p*	0.739	0.012		0.070	0.044		0.058	<0.001	
*Sfe/Sv*									
Acupuncture	0.075 ± 0.020	0.040 ± 0.022	46.399^***^	0,049 ± 0.025	0.035 ± 0.024	67.228^***^	0.122 ± 0.031	0.095 ± 0.032	75.114^***^
Rehabilitation Therapy	0.076 ± 0.019	0.063 ± 0.018	49.210^***^	0.047 ± 0.018	0.041 ± 0.019	65.576^***^	0.120 ± 0.026	0.102 ± 0.025	63.508^***^
t	0.300	10.132^***^		0.666	2.396^*^		0.352	2.147^*^	
*p*	0.765	<0.001		0.506	0.017		0.725	0.033	
*Sfp/S*_v_									
Acupuncture	0.029 ± 0.008	0.024 ± 0.008	17.095^***^	0.026 ± 0.005	0.021 ± 0.005	124.818^***^	0.029 ± 0.010	0.025 ± 0.010	38.704^***^
Rehabilitation Therapy	0.030 ± 0.005	0.026 ± 0.004	34.902^***^	0.025 ± 0.004	0.022 ± 0.004	47.755^***^	0.030 ± 0.007	0.027 ± 0.006	23.051^***^
t	0.504	2.412^*^		1.583	2.503^*^		0.842	2.588^*^	
*p*	0.614	0.016		0.114	0.013		0.400	0.011	

*Sv,* Vertebral Area; *Sfm,* Multifidus Fat Infiltration Area; *Sfe,* Erector Spinae Fat Infiltration Area; *Sfp,* Psoas Major Fat Infiltration Area; *Sf/Sv,* Paravertebral muscle fat infiltration Area to vertebral area ratio; *Sfm/Sv,* Multifidus muscle fat infiltration Area to vertebral area ratio; *Sfe/Sv,* Erector spinae muscle fat infiltration Area to vertebral area ratio; *Sfp/Sv,* Psoas major muscle fat infiltration Area to vertebral area ratio. ***p<0.001, *p<0.05.

### Analysis of VAS and JOA scores before and after treatment

This study utilized the Visual Analogue Scale (VAS) to assess pain levels and the Japanese Orthopaedic Association (JOA) score to evaluate the quality of life in patients (**Table 4**). Pre-treatment, the VAS scores for back pain in the acupuncture and rehabilitation groups were (4.18 ± 0.90, 4.10 ± 0.94, p=0.404) and for leg pain were (4.16 ± 0.98, 4.17 ± 1.05, p=0.914). The JOA scores for the acupuncture and rehabilitation groups were (19.01 ± 1.54, 18.86 ± 1.61, p=0.385). There were no significant differences in VAS scores for back and leg pain or JOA scores between the groups (P>0.05), indicating comparable baselines for pain and quality of life. Two weeks post-treatment, the VAS scores for back pain in the acupuncture and rehabilitation groups decreased to (0.27 ± 0.44, 0.32 ± 0.47, p=0.278), and for leg pain to (0.41 ± 0.49, 0.46 ± 0.62, p=0.433). The JOA scores were (25.39 ± 1.14, 25.18 ± 1.21, p=0.113). At this stage, inter-group comparisons revealed no significant differences in VAS and JOA scores (P>0.05), indicating a comparable level of therapeutic effectiveness between the groups during the initial treatment phase. However, there was a significant improvement in these scores compared to pre-treatment (P<0.05), reflecting a considerable decrease in pain and enhancement in quality of life post-treatment. Three months post-treatment, the acupuncture and rehabilitation groups reported VAS scores for back pain as (0.15 ± 0.36, 0.28 ± 0.45, p=0.003) and for leg pain as (0.11 ± 0.32, 0.32 ± 0.47, p<0.001) respectively. The JOA scores were (26.75 ± 0.88, 26.55 ± 0.92, p=0.045). The acupuncture group showed more significant improvements in both VAS scores for back and leg pain and JOA scores compared to the rehabilitation group, with statistical significance (P<0.05), indicating a more pronounced effect of acupuncture treatment on pain reduction and quality of life enhancement. This suggests that acupuncture therapy was more efficacious in mitigating pain and elevating patients’ quality of life than the rehabilitation treatment. While the therapeutic outcomes appeared similar in the initial phase of treatment, long-term observations highlighted that acupuncture treatment yielded more significant improvements in both lumbar and leg pain, as well as in the overall quality of life.

**Table 4:** Secondary outcome comparisons between the acupuncture group and the rehabilitation therapy group (lumbar pain, leg pain VAS, and JOA scores).

Groups	Lumbar Pain VAS Score			Leg Pain VAS Score			JOA Score		
	Pre-treat	2w post-treat	3m post-treat	Pre-treat	2w post-treat	3m post-treat	Pre-treat	2w post-treat	3m post-treat
Acupuncture	4.18 ± 0.90	0.27 ± 0.44^∆^	0.15 ± 0.36^▲^	4.16 ± 0.98	0.41 ± 0.49^∆^	0.11 ± 0.32^▲^	19.01 ± 1.54	25.39 ± 1.14^∆^	26.75 ± 0.88^▲^
Rehabilitation Therapy	4.10 ± 0.94	0.32 ± 0.47^❑^	0.28 ± 0.45^★^	4.17 ± 1.05	0.46 ± 062^❑^	0.32 ± 0.47^★^	18.86 ± 1.61	25.18 ± 1.21^❑^	26.55 ± 0.92^★^
T	0.836	1.085	2.959	0.108	0.785	4.660	0.871	1.588	2.010
*P*	0.404	0.278	0.003	0.914	0.433	<0.001	0.385	0.113	0.045

∆❑▲★ compared to baseline, p<0.05, the difference is statistically significant.

## Discussion

In the present study, we embarked on an in-depth investigation of the therapeutic efficacy of acupuncture and rehabilitation treatments for LDH, as well as their impact on paravertebral muscles and fatty infiltration. Unlike many studies that focus solely on acupuncture (27), Our findings underscored that both treatments notably enhanced patients’ quality of life and ameliorated conditions of paravertebral muscles and fatty infiltration within a span of two weeks and three months post-treatment. While both treatments are effective, acupuncture shows statistically significant advantages in improving muscle indicators and pain relief over a longer term,. The association between lumbar disc protrusion and paravertebral muscles represents a nascent yet profoundly clinically relevant domain of research. Paravertebral muscles play a pivotal role in preserving spinal stability, being integral in both static and dynamic states. A well-functioning state of these muscles is imperative for maintaining spinal stability (39, 40). Muscle degeneration, manifesting as atrophy, fatty infiltration, alterations in muscle fiber types, and changes in muscle activity, can impair the biomechanics and motion of spinal units in patients with lumbar pain. Paraspinal muscles may be influenced by confounding factors such as age, gender, duration of illness, obesity, and smoking, which are prevalent in patients with Lumbar Disc Herniation (LDH). We adjusted for age, gender, ethnicity, and BMI in our model to exclude potential confounders. Recent studies suggest that muscles and bones share common genetic, nutritional, lifestyle, and hormonal determinants (41), with low muscle mass and strength depending on BMI (42), A lower BMI is associated with muscle loss, whereas a higher BMI is not (43). Exercise interventions have been shown to beneficially impact muscle quality, strength, and physical performance (44). Literature reports that hypertension, cognitive impairments, hyperlipidemia, heart disease, stroke, cancer, pain, anemia, and liver disease are not associated with an increased risk of muscle loss (43). Similarly, geographical differences relate to many important human health factors such as latitude, dietary variations, and hours of sunlight exposure. All these factors could directly or indirectly contribute to variations in muscle loss. Moreover, modifications in the structure and function of these muscles might be linked to the recurrence or chronicity of lumbar pain (45). Numerous studies have elucidated this association, with Denis’s 1983 proposition of the “Three-column theory of the spine” laying the foundational theoretical framework for understanding the integral role of paravertebral muscles in spinal stability. Lumbar pain induced by LDH frequently coincides with atrophy or functional impairments in paravertebral muscles. The anterior lumbar large muscle and the posterior extensor muscle group, encompassing the ES and MF, are paramount in ensuring lumbar stability (46). However, despite evidence pointing to marked atrophy in paravertebral muscles of patients with LDH, a comprehensive understanding of its etiology and implications remains in its infancy (47).

LDH is a common degenerative spinal disease in clinical practice, primarily manifesting as lumbar pain and radiating pain in the lower extremities. These symptoms not only severely impact patients’ daily activities but can occasionally lead to neurological dysfunctions, imposing significant economic burdens on families and the healthcare system (2). Recent global prevalence data indicate that the incidence of LDH is on the rise worldwide, particularly among younger populations (48–50). This trend is largely attributed to changes in modern lifestyles and work environments (51), such as prolonged sitting and lack of physical exercise. Studies suggest that approximately 80% of adults will experience at least one episode of lower back pain during their lifetime, with lower back pain being one of the most common symptoms of LDH (3, 34, 52). The socioeconomic impact of LDH is profound. Direct medical costs include surgery, medication, and physical therapy (1), but more significant are the indirect costs related to pain and functional impairments that lead to unemployment and decreased productivity (53). Chronic disability may result in long-term dependency on pain management and rehabilitation services, thus increasing the societal burden. It has been reported that lower back pain, including that caused by LDH, is one of the leading causes of work disability, placing immense pressure on the economy (54). Acupuncture, an ancient therapeutic technique, has been empirically shown to modulate endogenous pain regulatory networks, thereby offering analgesic benefits to LDH patients with lumbar and leg pain (55). Compared to other conservative treatment modalities, acupuncture has demonstrated superior efficacy in managing LDH (56), Although rooted in traditional practices, recent research underscores its significant therapeutic impact on LDH patients, mitigating lumbar pain, leg pain, enhancing lumbar mobility, and improving overall quality of life (57). Our study corroborates these findings, underscoring the remarkable efficacy of acupuncture in LDH management. We observed a tangible reduction in pain and localized tenderness among LDH patients subjected to acupuncture. Further accentuating our results, we noted a marked alleviation in lumbar pain post-treatment, irrespective of whether patients underwent acupuncture or rehabilitative therapy. This not only emphasizes the efficacy of both modalities but also suggests the potential synergistic effects when combined, warranting further exploration in future research endeavors.

A significant correlation exists between LDH and the degeneration and fatty infiltration of paraspinal muscles. Research indicates that severe fatty infiltration is associated with neural root compression, and the severity of this infiltration can escalate in the presence of such compression (58). Moreover, there are indications that patients with advanced disc degeneration exhibit increased chances of fatty infiltration in the MF and ES (59). In younger patients with lumbar disc herniation, an increased thickness of subcutaneous fat tissue at the L1-L2 level, prolonged extended symptom duration, and more pronounced atrophy in the normal side of the MF are reported to correlate with elevated occurrences of lumbar pain (14). Lee et al. discovered through MRI assessments that patients with lumbar flatback deformity demonstrated evident atrophy of the lumbar paraspinal muscles, characterized predominantly by reduced muscle volume and conspicuous intermuscular fat infiltration (60). In our study, we evaluated the fatty tissue area’s variations in the ES, MF, and PM relative to vertebral CSA pre- and post-treatment. Notably, our results indicated that the acupuncture treatment group exhibited a more pronounced reduction in muscle fat deposition compared to the rehabilitative therapy group. Acupuncture, a non-surgical intervention, involves needle insertion into muscle tissues followed by manipulative rotations. It has been substantiated to effectively ameliorate pain intensity and range of motion in LDH patients (61). Consistent with previous research, our results show significant pain relief and functional improvement three months post-treatment. Similar findings were reported by Zhong et al (26). The literature documents greater improvements with electroacupuncture and spinal manipulation in terms of pain in the lower back, buttocks, and legs, as well as associated disabilities (28, 62). However, our study further explores the timeline of these improvements, noting no significant differences at two weeks, but substantial improvements at three months, providing a more nuanced understanding of the temporal dynamics of acupuncture effects. Clinical trials have demonstrated that post-acupuncture, there’s an augmentation in local blood circulation and a rise in soft tissue temperature. This facilitates inflammation resolution, alleviates muscle spasms, fosters an increase in the volume of paraspinal muscles, and diminishes the proportion of fatty infiltration between them.

Acupuncture, a traditional Chinese medicinal technique, aims to modulate the body’s internal flow of Qi and blood by stimulating specific acupoints, thereby achieving therapeutic effects. The primary mechanisms underlying the localized pain of LDH originate from atrophy and excessive tension in the paraspinal muscles, combined with the occurrence of aseptic inflammation and spasms (63). These pathological changes are principally related to the diminished interaction between muscle and fascial structures, reductions in muscle quality, strength, and regenerative capacity, and the increase in fascial rigidity coupled with decreased elasticity. Furthermore, the atrophy of the motor cortex, stemming from aging, reduces its excitability and plasticity, thus accelerating the accumulation of denervated muscle fibers (64). Contemporary research has validated the efficacy of acupuncture in alleviating pain, enhancing sleep quality, and elevating overall life quality. Our study highlights the advantages of acupuncture in treating LDH and uncovers its significant role in mitigating paraspinal muscle atrophy among LDH patients. Our findings indicate an increase in the area ratio of multifidus (MF), erector spinae (ES), and psoas major (PM) relative to the vertebral body after acupuncture treatment, along with a reduction in fatty infiltration. This is consistent with literature studies (65–67), which emphasize the efficacy of electroacupuncture in enhancing muscle strength of LDH patients and reducing fat infiltration. However, our study provides additional insights into the differential responses of various muscle groups, which have not been extensively reported in earlier literature. It’s noteworthy that the improvements in the PM were not as pronounced as those in the ES and MF. This discrepancy might stem from the acupuncture treatment primarily targeting the latter two muscles, bypassing direct intervention on the psoas major. Nevertheless, with enhancements in the ES and MF, we anticipate improvements in the condition of the psoas major over time. Given our study’s short follow-up duration, noticeable improvements in the psoas major were not evident. Thus, our findings underscore acupuncture’s potential in notably ameliorating the atrophy in the ES and MF.

Acupuncture, an age-old therapeutic modality, has demonstrated potential in addressing LDH and the associated paravertebral muscle atrophy through multiple mechanisms. Primarily, HA-19, a derivative of oleanolic acid, has demonstrated significant potential in mitigating muscle atrophy by activating the mTORC1/p70 S6K pathway to enhance protein synthesis. It also upregulates myogenic transcription factors such as Pax7 and MyoD, which are critical for the proliferation and differentiation of myoblasts, thus effectively counteracting muscle atrophy (68). Additionally, acupuncture increases the expression of neurotrophic factors, including brain-derived and glial cell-derived neurotrophic factors, which are essential for nourishing peripheral nerves. Moreover, acupuncture elevates levels of IGF-1, a potent agent for muscle regeneration, offering further resistance against muscle atrophy. It also reduces neuronal excitability within muscles, easing muscle and fibrous tissue tension. The increased localized temperature and enhanced blood flow induced by acupuncture contribute significantly to its therapeutic effects on paravertebral muscle atrophy. The selection of an optimal internal reference gene for muscle tissue continues to be a debated issue in the field. Tokłowicz et al (69) embarked on an evaluation of 90 tissue samples from deep and superficial paravertebral muscles of the convex and concave sides of spinal curvatures. In their quest to assess the stability of 12 miRNAs, they discovered three with reference potential, while the remaining nine exhibited tissue-specific properties. While our findings bear significant clinical implications, this realm necessitates more in-depth exploration. It’s pivotal to unearth the precise roles of these mechanisms and fully realize their potential in therapeutic contexts.

This study introduces a novel perspective in understanding the etiology of LDH, emphasizing the pivotal role of paravertebral muscle function. Notably, if targeted interventions for these muscles were initiated prior to the diagnosis of LDH, or even before its onset, it might present an opportunity to prevent or at least delay the progression of the condition. Consequently, future investigations should delve deeper into the causative relationship between paravertebral muscle atrophy and LDH, offering fresh avenues and evidence for preventive strategies. However, our research is not without limitations. The relatively small sample size necessitates larger and more rigorous prospective studies to ensure objectivity and accuracy. Additionally, while our participants primarily came from several hospitals in China and the acupuncture techniques and specific points used in our study are standardized, making them highly reproducible and applicable across different regions or countries, variations in the practice of acupuncture points and techniques in other regions or countries might affect our results. Moreover, the follow-up period of our study was three months, primarily to observe the short-term effects post-treatment. This brief follow-up period only allows for the assessment of short-term therapeutic effects, overlooking potential long-term treatment outcomes. Considering these factors, although our findings are useful, they underscore the need for extensive long-term studies to further validate and establish these preliminary insights. Future studies should further explore the long-term effects of acupuncture on the morphology and fat infiltration of paravertebral muscles and evaluate its effectiveness in preventing the recurrence of lumbar disc herniation (LDH). Additionally, it is advisable to conduct more randomized controlled trials to verify the effects of acupuncture across different LDH patient groups, thereby optimizing and personalizing acupuncture treatment strategies to enhance treatment efficacy and patient satisfaction. Prospective studies are also recommended to validate our findings, investigate the long-term effects of acupuncture on muscle health, and compare different acupuncture techniques. Finally, molecular mechanism studies at the cellular and molecular levels should be conducted to deeply understand how acupuncture affects paravertebral muscles at the biological level.

## Conclusions

Acupuncture treatment for LDH has been shown to effectively increase the CSA of the MF, ES, and PM. Additionally, it ameliorates paravertebral muscle atrophy and reduces fat infiltration in these muscles. Concurrently, patients experience notable relief in pain across the lumbar and leg regions and exhibit a marked improvement in their overall quality of life. These findings robustly advocate for the inclusion of acupuncture in clinical practices treating such conditions.

## Data Availability

The original contributions presented in the study are included in the article/supplementary material. Further inquiries can be directed to the corresponding authors.
